# Neuromodulation of Nestmate Recognition Decisions by Pavement Ants

**DOI:** 10.1371/journal.pone.0166417

**Published:** 2016-11-15

**Authors:** Andrew N. Bubak, Jazmine D. W. Yaeger, Kenneth J. Renner, John G. Swallow, Michael J. Greene

**Affiliations:** 1 Department of Integrative Biology, University of Colorado – Denver, Denver, Colorado, United States of America; 2 Neuroscience Program, University of Colorado – Anschutz Medical Campus, Denver, Colorado, United States of America; 3 Department of Biology, University of South Dakota, Vermillion, South Dakota, United States of America; University of Mississippi, UNITED STATES

## Abstract

Ant colonies are distributed systems that are regulated in a non-hierarchical manner. Without a central authority, individuals inform their decisions by comparing information in local cues to a set of inherent behavioral rules. Individual behavioral decisions collectively change colony behavior and lead to self-organization capable of solving complex problems such as the decision to engage in aggressive societal conflicts with neighbors. Despite the relevance to colony fitness, the mechanisms that drive individual decisions leading to cooperative behavior are not well understood. Here we show how sensory information, both tactile and chemical, and social context—isolation, nestmate interaction, or fighting non-nestmates—affects brain monoamine levels in pavement ants (*Tetramorium caespitum*). Our results provide evidence that changes in octopamine and serotonin in the brains of individuals are sufficient to alter the decision by pavement ants to be aggressive towards non-nestmate ants whereas increased brain levels of dopamine correlate to physical fighting. We propose a model in which the changes in brain states of many workers collectively lead to the self-organization of societal aggression between neighboring colonies of pavement ants.

## Introduction

Most ant colonies are closed societies from which conspecific and heterospecific intruders are excluded from a colony’s territory because of worker aggression towards non-nestmates [1–4]. When ants interact, they touch antennae to the other ant’s body and assess local information cues that are present in the mixture of long-chain cuticular hydrocarbon molecules [3–5]. Information coded in cues is assessed to determine if a contacted ant has nestmate or non-nestmate membership. Identification and exclusion of territorial intruders is important because colony survival depends upon food collection in competition with neighbors [6–8]. Colony behavior self-organizes as a result of many individual decisions, solving complex problems such as engaging in aggressive social conflicts with neighboring societies or discriminating between food sources of opposing value [9–14].

The pavement ant, *Tetramorium caespitum* (subfamily Myrmicinae), is a tramp ant species abundant in Northern Temperate urbanized and agricultural habitats [15–17]. Pavement ant workers perform random walks to search colony territory for foods high in sugar and fat content [13; 18]. In order to secure territory boundaries, pavement ant workers organize conspicuous wars, involving thousands of ants, against neighboring colonies [19–20]. In these wars, fighting is ritualized and few ants die [20]. Most workers fight in pairs, or dyads, grasping mandibles or other body parts and remain engaged for up to 12 hours. A worker is likely to fight a non-nestmate ant if it antennates the other ant, detects mis-matches in nestmate chemical recognition cues, and has had a recent history of interaction with nestmate ants [19]. Other ants recruit workers from colonies to the war using pheromone trails and wars establish quickly, within about 30 minutes [20]. Pavement ant wars are likely ritualized tournaments to display colony size at territorial boundaries [19–20].

The monoamines serotonin (5-HT), dopamine (DA), and octopamine (OA), the invertebrate analogue to norepinephrine, regulate specific individual behaviors in a variety of invertebrate and vertebrate species [21–32]. For social insects, the monoamines modulate many behaviors important to ant colony function, including colony formation, reproductive dominance, division of labor, behavioral development, trophallaxis, predatory aggression, trail following, and nestmate recognition [21–32]. For example, neural 5-HT and DA have been shown to modulate social food flow among workers using trophallaxis as well as division of labor [21–23]. Additionally, OA has been shown to enhance nestmate recognition acuity in honeybees and fire ants, *Solenopsis invicta*, as well as predatory aggression in the Australian weaver ant, *Oecophylla smaragdina* [30–32]. However, in social insects, the causal relationships between the monoaminergic systems and how they might modulate appropriate organized behavioral responses to specific social contexts still remains largely undetermined. If social interactions among individuals lead to monoaminergic synchronization and behavioral changes, then coordinated, collective behavior, as proposed previously [21], can result.

An agent-based model describing decisions to fight by pavement ants provides evidence that interactions with nestmate ants, prior to interacting with a non-nestmate, leads to increases in the monoamines 5-HT and OA; when levels exceed a threshold, a worker is likely to fight a non-nestmate ant [19]. Here we present a novel neurophysiological mechanism employed by pavement ants that leads to collective organization into aggressive competitions with neighboring societies. Much like a set of switches, the monoamines 5-HT, DA, and OA differentially modulate individual decisions based on context-specific information gathered by local cues.

## Materials and Methods

### Collection of ants

Pavement ant workers were collected in central Denver, Colorado, U.S.A., from colonies separated by at least 3 m, a reliable distance between separate colonies. Ants were collected by aspiration from foraging trails leading to food baits. Pavement ants are easily recruited to food that is high in sugar or fat [13]. Ants were collected on public sidewalks and streets; therefore, no specific permissions were required for collection. No endangered or protected species of animals were used in the studies. Ants were used in experiments usually within 24 hours. Ants were temporarily housed at room temperature (25°C) in plastic containers and allowed to drink *ad libitum* from glass tubes filled with water and plugged with cotton.

### Chemical Methods

Ant mimics were created by extracting cuticular hydrocarbons from ants and transferring them to 2 mm glass beads (Fisher Scientific). Cuticular hydrocarbons were extracted using standard methods [33–35]. Surface lipids were extracted from thawed ants that were killed by freezing at -20°C, by soaking them in 1 mL of 100% pentane (Aldrich Chemicals) for 10 minutes with periodic shaking. Long-chain hydrocarbons are unreactive and not susceptible to evaporation in storage; freezing does not significantly cause quantitative or qualitative changes in cuticular hydrocarbon profiles compared to fresh samples. Cuticular hydrocarbons were separated from more polar surface lipids by eluting extracts with 2 mL of 100% pentane through a 2 cm column of silica gel (Merck; grade 60, 70–230 mesh, 60Å) in a 5.75 inch glass Pasteur pipette. The extracts were added to a glass tube containing 2 mm glass beads equal in number to the ants extracted. The solvent was allowed to evaporate thus transferring one ant-equivalent of purified cuticular hydrocarbons from ants to each ant mimic.

### Brain Biogenic Amine Sample Preparation and Dissection

Ants were rapidly decapitated with micro-scissors and prepped for brain removal following previous methods conducted on other small insects [26–27, 36]. Heads were visualized under a dissection microscope and held stationary by micro-tweezers. A small medial-lateral incision was made directly behind the mandibles as to not disrupt brain tissue. The dorsal portion of the exoskeleton was pulled from the rest of the head, rostral to caudal, using micro-tweezers to expose the inside of the head capsule. Neural tissue was then removed with tweezers and submerged in 60 μL of ice-cold acetate buffer containing the internal standard, alpha-methyl DA. Samples were placed on dry ice and stored at -80°C until biochemical quantification by high performance liquid chromatography with electrochemical detection (HPLC). Dissection time averaged less than one minute per ant. Samples contaminated with pieces of cuticle, which can affect DA levels, were not analyzed by HPLC [36].

We pooled two brains per sample for chemical analysis, a common method for insect samples, because pavement ants have brains of small volume and a sample of two brains allowed the detection of monoamine peaks that exceeded a 2:1 signal to noise ratio in the chromatograms [35]. Ants were selected randomly for dissection and sample pooling.

### Biochemical Quantification of Brain Biogenic Amines

The biogenic amines OA, DA, and 5-HT were quantified using high performance liquid chromatography with electrochemical detection following a previously developed method with slight modifications [26–27; 35]. The brain samples were thawed, briefly sonicated and centrifuged at 17,000 rpms and 50 μL of the supernatant was injected into a Waters Alliance e2695 separations module. The amines were separated using a C18 4 μm NOVA-PAK radial compression column (Waters Associates, Inc. Milford, MA). The mobile phase consisted of 8.6 g sodium acetate, 250 mg EDTA, 14 g citric acid, 130 mg octylsulfonic acid, and 160 mL of methanol, pH 4.1 (all chemicals were obtained from Sigma-Aldridge, St. Louis, MO) in 1 L distilled water. The monoamines were detected electrochemically using an LC 4 potentiostat and glassy carbon electrode (Bioanalytical Systems, West Lafayette, IN) set at a sensitivity of 0.5 nA/V with an applied potential of +0.975 V versus an Ag/AgCl reference electrode. The tissue pellet was dissolved in 100 μL of 0.4M NaOH and analyzed for protein to correct for tissue volume [36–37]. The brain monoamine concentrations were calculated using peak height values obtained from OA, DA and 5-HT standards (Sigma-Aldridge, St. Louis, MO) with a CSW32 data program (DataApex Ltd., Czech Republic) set to the internal standard mode. All samples contained the internal standard alpha-methyl DA. Appropriate corrections for injection volume vs preparation volume were made and the resulting amine concentrations were divided by μg protein in the sample to yield pg amine/μg protein. Sample sizes for brain monoamine analysis differed due to technical problems that resulted in background noise that exceeded the signal in certain chromatograms.

### Behavioral tests

Experiments were conducted in the laboratory by placing ants into Petri dish arenas. At the beginning of a trial, a smaller, bottomless 60 mm diameter dish with Insect-a-Slip (Bioquip) treated sides was placed into a larger, 100 mm diameter dish, also with Insect-a-Slip treated sides, and a bottom. After 15 minutes, during which nestmates were allowed to interact, the inner arena wall was removed and ants from the two colonies were allowed to interact and fight. All measurements of fighting were performed by a blind observer who was naïve to the experimental treatments. Colonies were tested once in all experiments and no ants were used more than once in experiments.

### Density-dependent response

To assess whether the decision to fight is density dependent, we placed a different densities of nestmate ants to non-nestmate ants in the inner and outer arenas respectively: 1:1, 2:2, 3:3, 4:4, 5:5, 6:6, 7:7, 8:8, 9:9, and 10:10. We chose a range of 1:1 to 10:10 because previous observations had shown that non-nestmate ants paired in a 1:1 density rarely fight while non-nestmate ants paired in a density of 10:10 commonly fight. We allowed nestmates to interact for 15 minutes and then we lifted the inner arena wall, allowing all ants to interact. After 3 minutes, we quantified the number of ants fighting in the arena. Once they begin, workers fight in pairs for many hours. Ants from a total of 18 replicate colonies were used in the experiment.

To determine how nestmate density corresponded to interaction rate, we placed 2, 3, 4, 5, 6, 7, 8, 9, and 10 nestmates in the small arena and sampled a randomly chosen ant (n = 7 ants from 7 replicate colonies), measuring the number of times they contacted another ant in 3 minutes to determine the mean interaction rate at each density of ants. We calculated density by dividing the number of ants in the arena by its area.

### Social context and brain monoamine levels

Twelve ants were placed into the smaller arena and 12 ants were placed in the larger arena. The ants in each arena were allowed to interact with nestmates for 15 minutes then the smaller arena wall was removed to allow the ants from the two arenas to interact. After 3 and 120 minutes, ants were collected for brain dissection. The 3 and 120-minute time points were designed to assess monoamine levels both early and late in social interactions or fights, as pavement ants fight wars for several hours.

We compared 5-HT, DA, and OA levels in ant brains that were a. isolated (one ant in outer arena); b. allowed to interact with live nestmates; and, c. allowed to fight with non-nestmate ants. Twelve isolated ants were placed in individual small arenas for 18 minutes (15 minutes + 3 minutes) or 135 minutes without any other ants. In another treatment, focal ants were allowed to interact with nestmates for 15 minutes before the inner arena wall was removed. They were then allowed to interact with nestmate ants for 3 minutes or 120 minutes until they were removed for brain dissection. For ants that were allowed to interact with non-nestmate ants, 12 unmarked ants from one colony were allowed to interact for 15 minutes in one arena while 12 marked non-nestmate ants were allowed to interact for 15 minutes in the other arena; after the inner arena wall was lifted, ants were allowed to fight for 3 minutes or 120 minutes. Only non-marked ants fighting at the time of brain dissection were used for monoamine analysis and all ants were from different colonies. Ants were marked by placing a dot of oil-based paint on their gaster.

In the next experiment, we compared brain 5-HT, DA, and OA levels from ants that were allowed to interact with blank glass beads, glass beads coated with nestmate cuticular hydrocarbon, and glass beads coated with non-nestmate cuticular hydrocarbons. This experiment was designed to determine if monoamine levels change in response to detection of chemical nestmate recognition cues alone rather than on the detection of those cues during behavioral interactions with live ants. Ants were exposed to ant mimics, 2 mm diameter glass beads (Fisher Scientific) that were coated with cuticular hydrocarbons extracted from live ants. Individual ants were placed in a 100 mm diameter arena with walls coated with Insect-a-Slip to prevent escape. Isolated ants were placed in the arena with no beads. Ants exposed to nestmate cuticular hydrocarbons were placed individually in an arena with 12 mimics coated with nestmate cuticular hydrocarbons for either 3 minutes or 120 minutes when ants were collected for dissection. Ants exposed to non-nestmate cuticular hydrocarbons were placed individually in an arena with 12 mimics coated with non-nestmate cuticular hydrocarbons for either 3 minutes or 120 minutes when ants were collected for dissection. Ants from 12 replicate colonies were used in the experiment.

### Behavioral changes in response to cuticular hydrocarbon cues alone

Single ants (n = 10 ants from different colonies) were placed in each of the arenas with 12 ant mimics for 15 minutes. Ant mimics were removed as well as the inner arena wall. For 5 minutes, any fighting by a pair was measured. The treatments were: 1) ants in both arenas exposed to blank beads, 2) ants exposed to ant mimics coated with nestmate cuticular hydrocarbons, and 3) ants exposed to ant mimics coated with non-nestmate cuticular hydrocarbons.

### Manipulation of 5-HT and OA levels

Individual ants (n = 20 from different colonies) were isolated in disposable glass culture tubes for 60 minutes prior to treatment. Immediately following the isolation period, ants were placed in individual holding areas containing a cotton ball (1 cm diameter), soaked with the treatment solution (20 mg octopamine-hydrochloride (Sigma-Aldrich) in 1 mL of 10% sucrose solution) or (30 mg 5-hydroxytryptophan (5-HTP) (Sigma-Aldrich) in 1 mL of 10% sucrose solution). Control ants (n = 20 ants) were allowed to drink from a cotton ball soaked in 1 mL of 10% sucrose.

Ants were allowed to drink for 20 minutes before being placed in the trial arena where they interacted with a non-nestmate ant. In experiments, we only used ants that were observed to drink and had protruded gasters. Ants were paired from non-nestmate ants that were also treated with monoamines or control sucrose solution. After 5 minutes of interactions, an observer blind to treatment measured the number of ants fighting. We compared the proportion of ants fighting between treatment and control groups using a contingency table followed by a Chi-square test (Bonferroni’s adjustment α = 0.009).

We also dissected brains of ants from each of the control and monoamine treatments to confirm increases in brain 5-HT and OA concentrations. Mean brain 5-HT increased in individuals treated with 5-HTP (13 ± 0.93 pg/μg protein; n = 11, from different colonies) compared to controls (7.2 ± 0.31 pg/μg protein; n = 9 from different colonies). Likewise, OA was also increased following treatment with OA (151.9 ± 31.7 pg/μg protein; n = 9) compared to controls (14.2 ± 3.72 pg/μg protein; n = 9 all from different colonies).

## Results and Discussion

**Fig 1:**
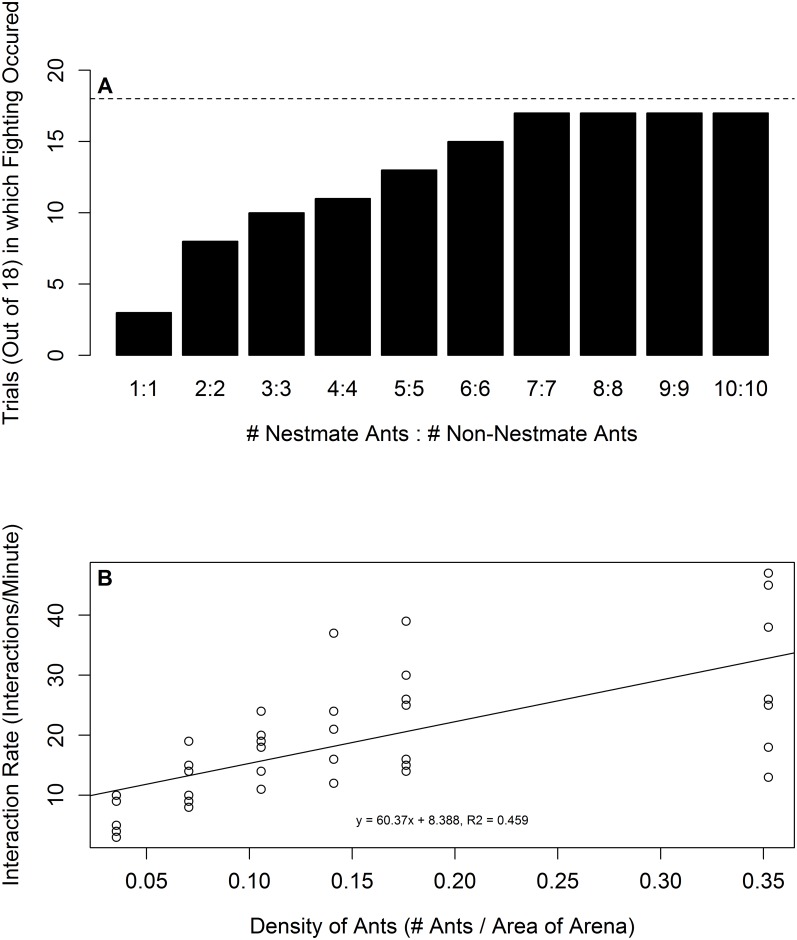
A) Pavement ant workers responded with fighting after exposure to non-nestmate ants in a density dependent manner (Beta regression; p < 0.0001; n = 18). Isolated ants were unlikely to fight non-nestmate ants and the proportion of trials in which fighting occurred increased with ant density. B) Pavement ant workers increased their interaction rate with nestmates as density increased. (Linear regression; R^2^ = 0.459, p < 0.0001; n = 7).

A pavement ant worker will decide to fight a non-nestmate ant if two conditions are met: 1) there is a mismatch between information detected in cuticular hydrocarbon nestmate recognition cues and a template and 2) the ant has had a recent history of interactions with its fellow nestmates [19]. The probability that any pavement ant fought a non-nestmate increased with the density of nestmates (beta regression; p < 0.0001; n = 18; Fig 1A). The mean proportion of ants fighting also increased with density (Chi-squared = 45.629, df = 9, p < 0.0001). Isolated ants rarely fought non-nestmates (mean proportion of ants that fought (± SD; n = 18 trials) = 0.167 ± 0.38). In contrast, ants allowed to interact with nestmates engaged non-nestmate ants in fights more frequently (Table 1).

**Table 1:** The proportion of ants fighting increases with density of nestmates (n = 18/group). The mean proportions reported are number of fights per ant.

**Density**	**1:1**	**2:2**	**3:3**	**4:4**	**5:5**
Mean ± SD	0.167 ± 0.38	0.26 ± 0.35	0.27 ± 0.29	0.36 ± 0.32	0.39 ± 0.29
**Density**	**6:6**	**7:7**	**8:8**	**9:9**	**10:10**
Mean ± SD	0.44 ± 0.30	0.52 ± 0.32	0.64 ± 0.31	0.63 ± 0.27	0.69 ± 0.29

As the density of ants increased so did their interaction rate with nestmates (linear regression; y = 69.37x + 8.388, R² = 0.459, p < 0.0001; Fig 1B). An increased rate of interaction with nestmates indicates there is a high density of ants in the near neighborhood and that the territory should be defended; animals are often more likely to fight at higher densities [6; 38–39]. A low rate of interaction representing a low density of ants in the near neighborhood prevents the positive escalation of wars when those wars would yield costs to the colony.

**Fig 2:**
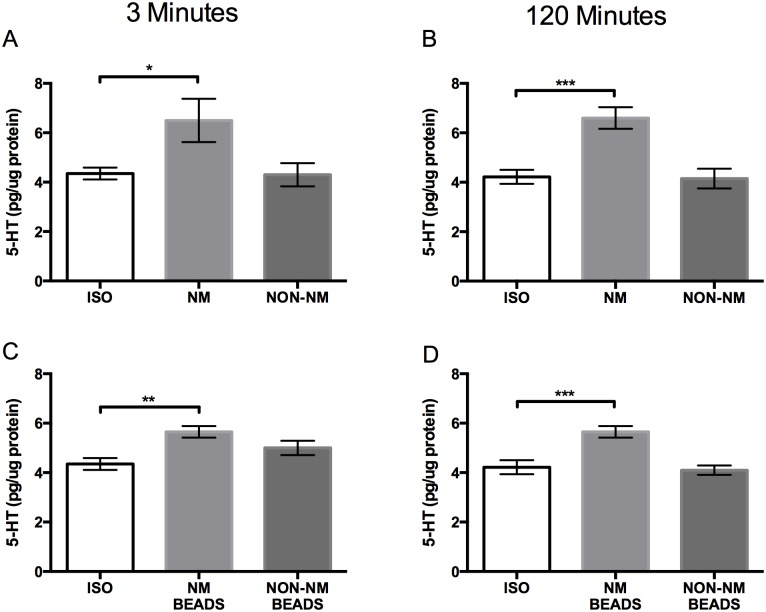
Top panels: Differences in brain 5-HT concentrations between isolated ants (ISO), ants that interacted with live nestmates (NM), and ants that interacted with live non-nestmates (NON-NM) for A) 3 minutes (n = 12 for ISO; n = 11 for NM; n = 9 for Non-NM) and B) 120 minutes (n = 12 for ISO; n = 11 for NM; n = 12 for Non-NM). Bottom panels: differences in brain 5-HT concentrations between isolated ants (ISO), ants that interacted with beads coated in nestmate hydrocarbons (NM BEADS), and ants that interacted with beads coated in non-nestmate hydrocarbons (NON-NM BEADS) for C) 3 minutes (n = 12 for ISO; n = 12 for NM; n = 12 for Non-NM) and D) 120 minutes (n = 12 for ISO; n = 12 for NM; n = 12 for Non-NM). Data reported as mean pg amine/μg protein ± SEM (Dunnett’s test; p < 0.05 *, p < 0.01 **, p < 0.001 ***).

**Fig 3:**
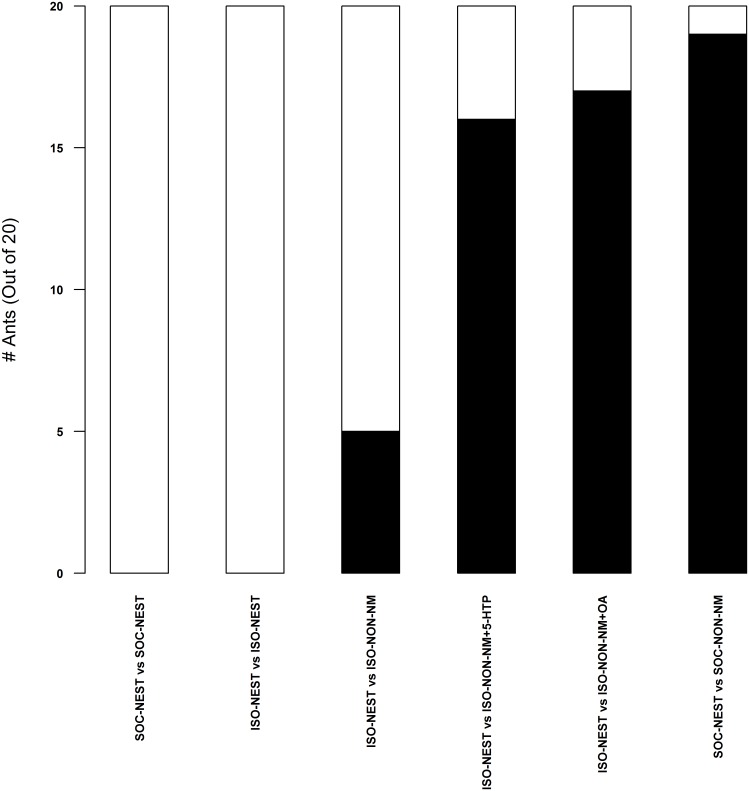
Pharmacologically increasing brain concentrations of 5-HT and OA (ISO-NON-NM+5-HTP and ISO-NON-NM+OA) increased fighting levels by isolated (ISO-NEST) ants (n = 20), which lacked a recent history of interaction with nestmates, when they met isolated non-nestmate ants (ISO-NON-NM). Black filled bars indicate ants that fought. Unfilled bar indicate ants that did not fight. SOC-NEST are ants that were allowed to interact with nestmates before interacting with nestmate ants in the assay. SOC-NON-NM were ants that were also allowed to interact with nestmates before interacting with non-nestmate ants. Contingency table with a Chi-square test, p < 0.001, df = 75.80, 5; Bonferroni’s correction (α = 0.009).

**Fig 4:**
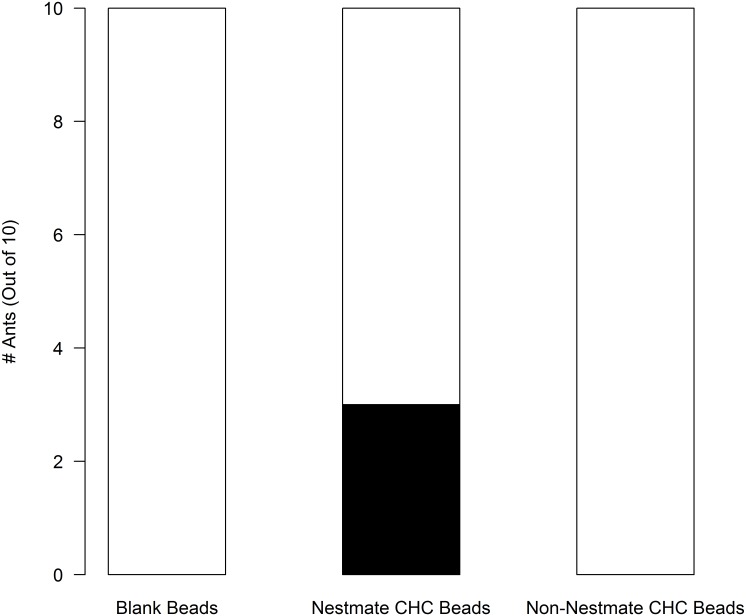
Interactions with ant mimics coated with nestmate cuticular hydrocarbons increased fighting when introduced to a non-nestmate ant (Contingency Table; Chi-square, p < 0.036, df = 6.667, 2; n = 10). Black filled bars indicate ants that fought. Unfilled bar indicate ants that did not fight.

In pavement ants that interacted with either live nestmates or ant mimics coated with nestmate cuticular hydrocarbon cues, brain 5-HT levels were elevated after 3 min and 120 min of exposure when compared to isolated ants (Fig 2). In contrast, 5-HT levels in ants that fought non-nestmates or interacted with non-nestmate cuticular hydrocarbons were unchanged compared to levels in brains from isolated ants (Fig 2). Furthermore, pharmacologically increasing brain 5-HT increased the levels of aggression displayed by individual ants that did not have a recent history of interactions with nestmates while isolated control ants seldom fought (Fig 3). These results suggest that 5-HT, which increases with interaction with nestmates or nestmate cuticular hydrocarbons, acts to prime pavement ant brains for fighting non-nestmate ants [19]. Ants that interacted with ant mimics coated with nestmate cuticular hydrocarbons also had elevated brain 5-HT levels (Fig 2C and 2D) and, after exposure, exhibited increased aggression towards non-nestmate ants (Fig 4).

The data in Table 1 show that, on a per ant basis (reported as proportions), fighting increased as nestmate density increased. Thus, the effect we report in Fig 1 and Table 1 was not simply due to having an increased number of ants, each with its own probability of fighting, but was instead best interpreted to be caused by the density of nestmate ants prior to exposure to non-nestmate ants. The data in Fig 4 also lend support this point. Here, isolated ants were exposed to isolated non-nestmate ants after a 15 minute exposure to ant mimics coated with nestmate, non-nestmate, or blank beads; isolated ants exposed to the nestmate mimics were more likely to fight a single non-nestmate ant. These data support the conclusion that the ants are using the rate at which they detect nestmate recognition cues coded cuticular hydrocarbons, as a measure of nestmate density. Although the ants were not tested at different nestmate densities of live ants, as in Table 1 and Fig 1, the experiment both controlled for the number of ants for which aggression was measured and focal ants were exposed to different densities of cues that code for nestmates, a proxy for live ants.

**Fig 5:**
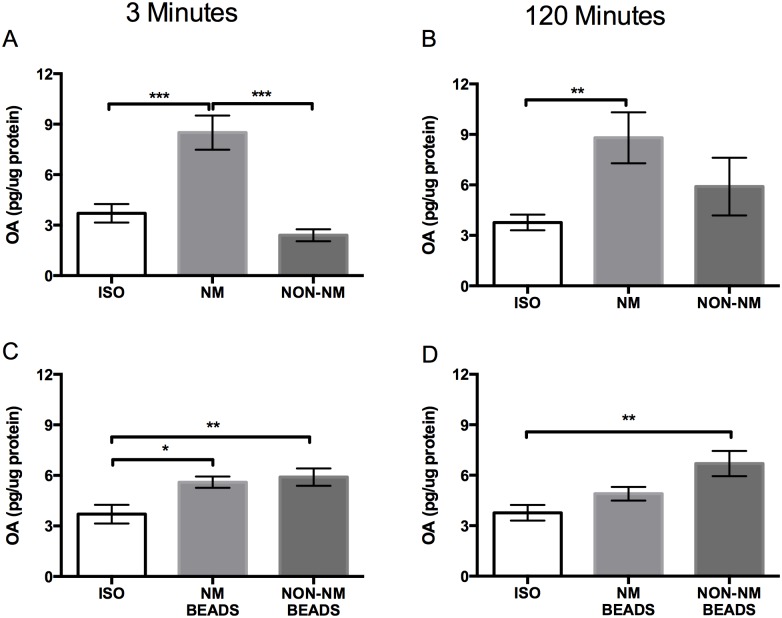
Top panels: Differences in brain OA concentrations between isolated ants (ISO), ants that interacted with live nestmates (NM), and ants that interacted with live non-nestmates (NON-NM) for A) 3 minutes (n = 9 for ISO; n = 7 for NM; n = 5 for Non-NM) and B) 120 minutes (n = 10 for ISO; n = 8 for NM; n = 5 for Non-NM). Bottom panels: differences in mean neural OA concentrations between isolated ants (ISO), ants that interacted with beads coated in nestmate hydrocarbons (NM BEADS), and ants that interacted with beads coated in non-nestmate hydrocarbons (NON-NM BEADS) for C) 3 minutes (n = 9 for ISO; n = 12 for NM; n = 11 for Non-NM) and D) 120 minutes (n = 10 for ISO; n = 12 for NM; n = 11 for Non-NM). Data reported as mean pg amine/μg protein ± SEM. (Dunnett’s test; p < 0.05 *, p < 0.01 **, p < 0.001 ***).

Brain concentrations of OA also rose significantly following live nestmate interactions but not after fights with non-nestmates (Fig 5A and 5B). However, unlike 5-HT, when ants were presented with ant mimics coated with nestmate hydrocarbons, OA levels only increased slightly after 3 min of exposure but were not elevated after the 120 min exposure (Fig 5C and 5D). In contrast, when ants were exposed to non-nestmate hydrocarbons, a longer lasting increase in OA was observed suggesting a role in non-nestmate recognition (Fig 5C and 5D). When we pharmacologically increased OA in isolated individual ants without a recent history of interactions with nestmates, they were more likely to fight non-nestmate ants than isolated controls (Fig 3). Together, these results support an agent-based model reported in [19] that, like 5-HT, increased brain OA levels above a threshold contribute to priming the ants to fight after receiving the necessary social context but, in addition, may also play a role in the recognition of non-nestmate hydrocarbons. The evidence suggests that this increase in OA concentration degrades once actual fighting begins (Fig 5A and 5B).

**Fig 6:**
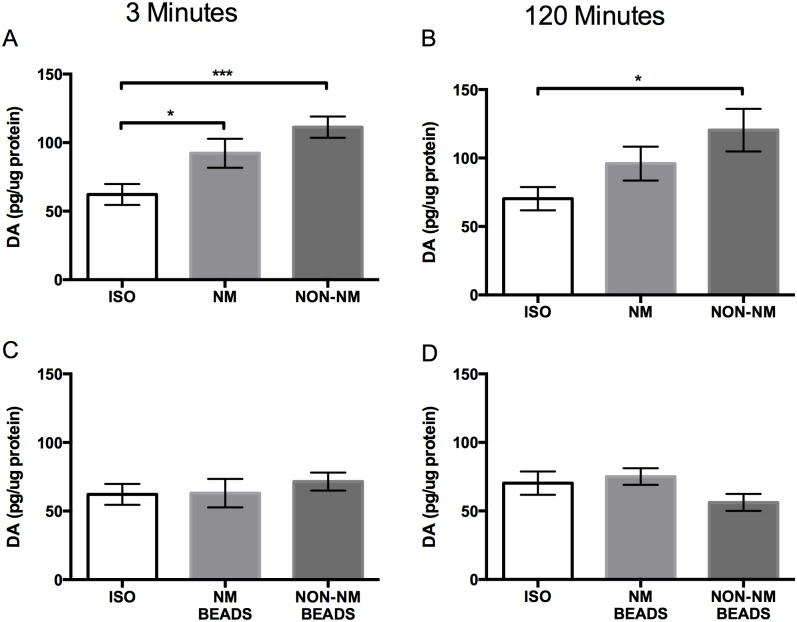
Top panels: Differences in mean neural DA concentrations between isolated ants (ISO), ants that interacted with live nestmates (NM), and ants that interacted with live non-nestmates (NON-NM) for A) 3 minutes (n = 12 for ISO; n = 12 for NM; n = 12 for Non-NM) and B) 120 minutes (n = 11 for ISO; n = 11 for NM; n = 8 for Non-NM). Bottom panels: differences in mean neural DA concentrations between isolated ants (ISO), ants that interacted with beads coated in nestmate hydrocarbons (NM BEADS), and ants that interacted with beads coated in non-nestmate hydrocarbons (NON-NM BEADS) for C) 3 minutes (n = 12 for ISO; n = 10 for NM; n = 12 for Non-NM) and D) 120 minutes (n = 11 for ISO; n = 12 for NM; n = 12 for Non-NM). Data reported as mean pg amine/μg protein ± SEM. (Dunnett’s test; p < 0.05 *, p < 0.01 **, p < 0.001 ***).

Dopamine levels were increased in brains of ants fighting live non-nestmate ants (Fig 6A and 6B). Dopamine levels did not increase with exposure to ant mimics coated with either nestmate or non-nestmate hydrocarbons indicating that this change is only associated with interactions between live ants, not with chemical cues presented on ant mimics (Fig 6C and 6D). Increased levels of DA at 3 minutes in brains from ants that interacted with live nestmates may be due to initial encounters with an unfamiliar nestmate or exposure to alarm pheromones since changes in DA are not evident when nestmates interacted for 120 minutes (Fig 6B). Pavement ants fight for hours during wars followed by an abrupt termination of the war signaled by unknown cues. Decreasing levels of brain DA during fighting may contribute to modulating this behavior. There appears to be a need for direct physical contact with other ants to elevate DA levels.

A pavement ant worker will make a decision to be aggressive during a nestmate recognition response if it has both a recent history of interactions with nestmates and recognizes a mismatch in cuticular hydrocarbons of a non-nestmate. Our results suggest a system in which 5-HT and OA levels increase during interactions with nestmates and DA levels increase in the brains of ants fighting non-nestmate ants. While relevant chemical cues are sufficient to increase 5-HT and OA levels in pavement ant brains, DA levels correlate to tactile stimulation during fighting, or the combination of tactile and chemical stimulation with non-nestmates during fighting. Unlike 5-HT, brain OA levels appear to change in response to exposure to unfamiliar cuticular hydrocarbon profiles, whereas changes in DA levels appear to be linked to physical contact with unfamiliar individuals during fighting. We suggest that such subtle differences can lead to a larger variety of behavioral decisions at the individual level, which, by extension, leads to collectively-organized behavior and self-organization at the colony level [21].

Our study has limitations, mainly that we conducted our experiments using queen-less laboratory fragments, as monoamine measurements were not possible from field sample. However, ants interact in a small-world, near-neighbor network and thus a member of a colony fragment should experience similar interaction patterns as those in a whole colony and, as a distributed system, the queen does not directly influence worker decisions [9]. Also, it is likely that other sources of information, such as tactile cues and trail pheromones, could alter monoamine levels and that monoamine levels, such as 5-HT, could influence the chemical detection of those pheromones [29]. Our data do not rule out those influences, but instead point to the specific influence of social interactions involving cuticular hydrocarbons on monoamine levels and aggressive behavior.

Our results show that when ants detect local nestmate recognition cues coded in cuticular hydrocarbons, these cues correlate with changes in brain monoaminergic function, which, in turn, modulates individual behavioral responses to social contexts. Furthermore, we suggest a model in which altering monoaminergic activity in the brain of individuals, thereby mimicking the neurochemical response to social cues, contributes to collective decision-making. Thus, we hypothesize, when ants encounter a nestmate or hydrocarbon cues from nestmates, the cues generate changes in monoaminergic function in individual brains that collectively affect a society’s decision to self-organize into an aggressive conflict with a neighbor. The subtle distinctions in serotonergic, octopaminergic, and dopaminergic activity in response to physical and non-physical cues presented in this study provide a well-refined mechanism for the complex network of colony behavior and collective decision-making.

## Supporting Information

S1 Data All data used for statistical analyses and figures for this paper. (XLSX)
